# Supplementary material for: In Vivo Targeted Metabolomic Profiling of Prostanit, a Novel Anti-PAD NO-Donating Alprostadil-Based Drug
Source: Molecules. 2020 Dec 13;25(24):5896. doi: 10.3390/molecules25245896 (PMC7764275; doi:10.3390/molecules25245896)
Supplement: Supplementary file 1 [file molecules-25-05896-s001.pdf]

Table S1. LC-MS\MS parameteres for Prostanite® metabolites

Chromatographic separation was conducted at 40°C using Acquity UPLC BEN C18 2.1 x 100 mm 1.7um. The mobile phases were 0.1% formic acid aqueous solution (solvent A) and acetonitrile with 0.1% formic acid (solvent B) with the following gradient program: 0 min (15% B), 15 min (60% B), 20 min (95% B), 25 min (95% B), 25.1 min (20% B), equilibration up to 30 min. Total run time for each analysis was 30 min with the injection volume of the sample 5 µl. The ionization method was employed in negative ESI mode. Capillary voltage was 2.5 kV, Source Temperature was 150°C, Desolvation GasTemperature was 600°C and Desolvation Gas Flow 1000 L/Hr. Multiple reaction-monitoring mode (MRM) parametres were in table S1.

	Retention Time	Precursor Ion	Product Ion	Cone Voltage	Collision Energy
1,3-DNG	3.75	227.1	62	15	5
Teriflunomide (IS)	10.25	269.1	82.1	30	20
PGB_1	11.90	335.2	317.2	35	22
PGA_1	11.63	335.3	317.3	35	15
15-keto-PGE_1	8.94	351.3	237.1	35	23
PGE_1	10.80	353.2	273.2	35	16
13,14-dehydro-15-keto-PGE_1	8.90	353.3	335.2	35	28
PGE_1-1,3-glycerol	3.75	473.2	391.1	20	20

Table S2. Retention time and MRM parameters for purine and pyrimidine profiling

Targeted determination of purine metabolites was carried according to parameters presented below. Chromatographic separation was operated at 40℃ using PFP Discovery® HS F5 HPLC Column 15cmx*2.1mm*3 µm (ThermoFinigan, USA) with mobile phases consisting of 0.1% formic acid aqueous solution (solvent A) and acetonitrile (solvent B). The gradient program was: 0-2 min (0%B); 2-5 min (25%B); 5-10 min (95%B); 10-15min (95%B); 15-15.1 (0%B); 15.1-22 (0%B); equilibration up to 22.1 min. Mass spectra were obtained at MRM mode (Supplementary Table 4). Capillary voltage was 1 kV, Source Temperature was 150°C, Desolvation Temperature was 500°C and Desolvation Gas Flow 1000 L/Hr.

	Retention Time	Precursor Ion	Product Ion	Cone Voltage	Collision Energy
γ-Aminobutyric acid	3,14	104	87	13	10
α-Aminobutyric acid	2.68	104	58	13	10
2-Aminoisobutyric acid	2.76	104	58	13	10
3-Aminoisobutyric acid	1.99	104	86	10	10
Uracil	5.80	113	70	26	15
Thymine	6.27	127	110	27	15
Ureidopropioic acid	2.90	133	115	11	10
Adenine	5.34	136	119	35	20
Guanine	4.55	152	135	25	18
Xanthine	4.25	153	110	30	15
Ureidosuccinic acid	2.57	117	136	5	10
Xanthurenic acid	7.83	206	132	10	15
Thymidine	6.27	243	127	18	13
Cytidine	4.94	244	112	23	12
Uridine	4.25	245	113	23	10
Deoxyadenosine	6.62	252	135	27	15
Adenosine	6.34	268	136	30	15
Deoxyguanosine	6.12	268	152	20	10
Inosine	5.85	269	137	20	15
Guanosine	5.94	284	152	18	20
Orotic acid	3.04	155	111	10	5
Uric acid	3.33	167	124	26	16
Deoxyuridine	5.80	227	184	22	12
Fluorouracil (IS)	4.65	131	114	15	15

Table S3. Retention time and MRM parameters for steroid profiling

Identification of steroid metabolites were made both in negative and positive ESI modes. Chromatographic separation was achieved at 40˚C using Waters ACQUITY UPLC CSH C18 column 1.7 µm (Waters, USA) with mobile phases consisting of 2.5 mM HCOONH4 aqueous solution (solvent A) and methanol (solvent B). The gradient program was as follows: 0.1 min – 30% B, 14 min – 90% B, 16 min – 90% B, 16,1 min – 30% B, equilibration up to 25 min with total run time 25 min. Injection volume of the sample was 3 µl. Mass spectra were obtained in MRM mode (Supplementary Table 3). Nitrogen was used as collision gas. Capillary voltage was 1 kV, Source Temperature was 150°C, Desolvation Temperature was 500°C and Desolvation Gas Flow 1000 L/Hr.

	Retention Time	Precursor Ion	Product Ion	Cone Voltage	Collision Energy
Melatonin	2.92	233	174.2	35	10
Androsterone	10.88	273.1	255.3	35	20
Androstendione	8.19	287.2	97	35	15
6-OH-Androsterone	8.07	289	151	35	10
DHEA	9.23	289.1	271.1	35	10
Testosterone	8.80	289.2	97	35	20
Methyltestosterone (IS)	9.43	303	97	35	20
Progesterone	10.60	315.1	97.2	35	20
Deoxycorticosterone	9.14	331.2	97.2	35	15
17-OH-Progesterone	7.35	331.2	97	35	20
Corticosterone	7.51	347.1	121.2	35	22
11-DC	7.35	347.2	97	35	25
21-DC	5.64	347.2	311.2	35	22
Cortison	6.16	361.2	163.1	35	20
Cortisol	8.09	363.2	121	35	24

Table S4. Retention time and MRM parameters for amino acid profiling

PFP Discovery® HS F5 HPLC Column 15cmx*2.1mm*3 µm (ThermoFinigan, USA) was used for chromatographic separation. The oven temperature was set at 40°C. The gradient elution involved a mobile phase consisted of 0.1% formic acid aqueous solution (solvent A) and acetonitrile (solvent B). The elution program was as follows: 0 -5min (0% B), then 15 min (40%), 20,1 min (100%), 25 min (100% B), 25.1 min (20% B), equilibration up to 35 min. Mass spectra were obtained at MRM mode with transitions presented in Supplementary Table 5. Capillary voltage was 1 kV, Source Temperature was 150°C, Desolvation Temperature was 500°C and Desolvation Gas Flow 1000 L/Hr.

	Retention Time	Precursor Ion	Product Ion	Cone Voltage	Collision Energy
Glycine	3.99	76	30	15	15
Alanine	2.20	90	44	15	12
Serine	2.01	106	60	15	13
Proline	2.52	116	70	15	15
Valine	3.71	118	72	15	12
Threonine	2.14	120	56	15	15
Pyroglu	3.67	130	84	17	15
4-Hydroxyproline	6.85	132	86	15	14
Leucine	6.53	132	86	15	12
Isoleucine	6.53	132	86	15	16
Ornitine	6.53	133	70	15	15
Aspartate	2.02	134	74	15	15
Lysine	2.10	147	84	15	17
Glutamate	2.20	148	84	15	15
Methioine	4.26	150	56	15	17
Histidine	2.50	156	110	15	15
Phenylalanine	8.82	166	120	15	17
1-Methylhistidine	2.54	170	126	15	15
Arginine	2.71	175	70	15	15
Tyrosine	6.32	183	81	15	29
DOPA	7.61	198	152	15	14
Tryptophan	6.28	206	188	15	10
Carnosine	8.85	227	110	15	25

Table S5. Validation parameters for the studied metabolites

To evaluate the matrix effect in the experiment, chromatographic peak areas of each analyte from the spike after extraction samples at low and high concentration levels were compared to the neat standards at the same concentrations. Percent nominal concentrations estimated were within the acceptable limits (86.7 – 101.5 %) after evaluating six different lots of plasma. The same evaluation was performed on IS and no significant peak area differences were observed. Thus, ion suppression or enhancement from plasma matrix was negligible for this method. Mean extraction recoveries were more than 94.6 % for all analytes. Observed effects were consistent and reproducible. The method showed good consistency throughout the entire standard concentration ranges.

Stability studies were performed for stock and working solutions of all analytes. The results revealed that all analytes were stable in stock and working solutions for 24 h at room temperature of about 25 ºC; for 60 h in the autosampler at 10 ºC and in refrigerator at +4 ºC – +8 ºC.

Target compound	Linearity (ng/mL)	R^2	LLOQ	Intra-day precision	Inter-day precision	% Recovery, ±RSD
1,3-DNG	0.5-100 ng/mL	0.9885	0.5	14.1	14.5	85
PGB_1	0.1-100 ng/mL	0.9923	0.1	10.2	9.8	91
PGA_1	0.1-100 ng/mL	0.9914	0.1	9.6	4.5	94
15-keto-PGE_2	0.1-100 ng/mL	0.9947	0.1	12.1	7.8	96
13.14-dihydro-15-keto-PGE_2	0.1-100 ng/mL	0.9915	0.1	5.6	6.5	92
PGE_2	0.1-100 ng/mL	0.9954	0.1	3.5	8.9	94
15-keto-PGE_1	0.1-100 ng/mL	0.9948	0.1	4.5	9.7	91
PGF_{2a}	0.1-100 ng/mL	0.9945	0.1	5.8	7.1	87
PGE_1	0.1-100 ng/mL	0.9919	0.1	4.5	6.3	93
13,14-dehydro-15-keto-PGE_1	0.1-100 ng/mL	0.9901	0.1	12.2	13.7	97
PGE_1-1,3-glycelol	0.5-100 ng/mL	0.9825	0.5	13.4	14.3	88
γ-Aminobutyric acid	0.2-100 ng/mL	0.9813	0.2	11.4	16.5	91

α-Aminobutyric acid	0.2-100 ng/mL	0.9942	0.2	10.1	11.4	94
2-Aminoisobutyric acid	0.2-100 ng/mL	0.9918	0.2	11.1	12.5	92
3-Aminoisobutyric acid	0.2-100 ng/mL	0.9923	0.2	12.4	13.1	93
Uracil	1-100 ng/mL	0.9814	1	12.66	13.1	93
Thymine	1-100 ng/mL	0.9942	1	11.56	12.6	91
Ureidopropioic acid	1-100 ng/mL	0.9912	1	9.14	8.6	93
Adenine	1-100 ng/mL	0.9923	1	4.5	5.1	99.
Guanine	1-100 ng/mL	0.9941	1	5.6	8.3	100
Xanthine	1-100 ng/mL	0.9947	1	14.2	11.8	94
Ureidosuccinic acid	1-100 ng/mL	0.9958	1	13.2	14.5	88
Xanthurenic acid	1-100 ng/mL	0.9946	1	11.8	12.4	87
Thymidine	1-100 ng/mL	0.9975	1	8.9	14.7	97
Cytidine	1-100 ng/mL	0.9975	1	5.6	12.0	98
Uridine	1-100 ng/mL	0.9815	1	11.3	11.5	97
Deoxyadenosine	1-100 ng/mL	0.9863	1	12.7	13.7	87
Adenosine	1 - 100 ng/mL	0.9845	1	13.5	11.0	96
Deoxyguanosine	1-100 ng/mL	0.9812	1	12.1	10.8	97
Inosine	1-100 ng/mL	0.9687	1	13.8	9.8	99
Guanosine	1 -100 ng/mL	0.9845	1	11.4	10.3	94
Orotic acid	10-1000 ng/mL	0.9824	1	5.1	6.3	95
Uric acid	10-1000 ng/mL	0.9848	1	4.8	5.9	96
γ-Aminobutyric acid	1-100 ng/mL	0.9948	1	10.2	10.6	85
Melatonin	0.05 -100 ng/mL	0.9912	0,05	12.8	14.8	89
Androsterone	0.01-100 ng/mL	0.9914	0.01	6.2	13.5	95
Androstendione	0.01-100 ng/mL	0.9855	0.01			

DHEA	0.01-100 ng/mL	0.9814	0.01	14.1	14.8	89
Testosterone	0.01-100 ng/mL	0.9985	0.01	6.8	7.0	94
Deoxycorticosterone	0.01-100 ng/mL	0.9942	0.01	7.2	8.1	95
17-OH-Progesterone	0.01-100 ng/mL	0.9947	0.01	4.1	5.6	98
Corticosterone	0.01-100 ng/mL	0.9946	0.01	8.6	13.1	89
11-DC	0.01-100 ng/mL	0.9913	0.01	3.8	7.5	95
21-DC	0.01-100 ng/mL	0.9921	0.01	4.6	9.1	93
Cortison	0.01-100 ng/mL	0.9984	0.01	1.2	6.5	98
Cortisol	0.01-100 ng/mL	0.9946	0.01	13.7	10.4	93
Glycine	1-1000 nmol/ml	0.9874	1	6.12	6.39	99
Alanine	1-1000 nmol/ml	0.9874	1	2.74	12.25	97
Serine	1-1000 nmol/ml	0.9914	1	1.13	12.53	97
Proline	1-1000 nmol/ml	0.9975	1	1.09	1.29	99
Valine	1-1000 nmol/ml	0.9846	1	2.18	5.40	98
Threonine	1-1000 nmol/ml	0.9845	1	6.53	4.30	99
Pyroglu	1-1000 nmol/ml	0.9845	1	6.74	9.66	97
4-Hydroxyproline	1-1000 nmol/ml	0.9913	1	8.86	4.33	103
Leucine	1-1000 nmol/ml	0.9844	1	5.51	6.06	96
Isoleucine	1-1000 nmol/ml	0.9913	1	4.18	2.24	94
Ornitine	1-1000 nmol/ml	0.9988	1	9.82	7.09	105
Aspartate	1-1000 nmol/ml	0.9989	1	2.29	4.49	104
Lysine	1-1000 nmol/ml	0.9933	1	2.77	4.67	98
Glutamate	1-1000 nmol/ml	0.9936	1	5.44	12.54	93

Methioine	1-1000 nmol/ml	0.9979	1	6.56	9.27	92
Histidine	1-1000 nmol/ml	0.9965	1	4.86	2.07	94
Phenylalanine	1-1000 nmol/ml	0.9914	1	5.43	11.75	95
Arginine	1-1000 nmol/ml	0.9814	1	13.49	7.65	103
Tyrosine	1-1000 nmol/ml	0.9923	1	6.40	7.62	108
DOPA	1-100 nmol/ml	0.9945	1	8.86	4.33	95
Tryptophan	1-1000 nmol/ml	0.9807	1	5.51	6.06	94
Carnosine	1-1000 nmol/ml	0.9801	1	4.18	2.24	88

Table S6. The reaction of Prostanit® and PGE_1 with NO detection reagents. 35 μM of Prostanit or PGE1 were incubated either with cell culture medium, or with PBS with 130 mM of NaBH4 for 10 min, after which the concentration of NO_2^- was determined using the Griess reaction (N=3 experiments). *, a statistically significant difference from cell culture medium; **, a statistically significant difference from Prostanit without cells, ANOVA with the Tukey post-test, $p<0.05$.

	Optical Density of the Griess Reaction, mean±S.E.
Cell culture medium	0.069±0.003
PGE_1	0.077±0.002 *
Prostanit	0.081±0.001 *
Prostanit +cells	0.898±0.011 *, **
Prostanit+$NaBH_4$	0.082±0.002 *
